# Mean field analysis gives accurate predictions of the behaviour of large networks of sparsely coupled and heterogeneous neurons

**DOI:** 10.1186/1471-2202-15-S1-O3

**Published:** 2014-07-21

**Authors:** Wilten Nicola, Felix Njap, Katie Ferguson, Frances Skinner, Sue Ann Campbell

**Affiliations:** 1 Department of Applied Mathematics & Centre for Theoretical Neuroscience, University of Waterloo, Waterloo, ON, Canada; 2 Division of Fundamental Neurobiology, Toronto Western Research Institute, Toronto, ON, Canada; 3 Department of Medicine (Neurology), University of Toronto, Toronto, ON, Canada; 4 Department of Physiology, University of Toronto, Toronto, ON, Canada

## 

Large networks of integrate-and-fire (IF) model neurons are often used to simulate and study the behaviour of biologically realistic networks. However, to fully study the large network behaviour requires an exploration of large regions of a multidimensional parameter space. Such exploration is generally not feasible with large network models, due to the computational time required to simulate a network with biologically significant size. To circumvent these difficulties we use a mean-field approach, based on the work of [1].

We consider a sparsely coupled, excitatory network of 10,000 Izhikevich model neurons [2], with Destexhe-type synapses [3]. The cellular models were fit to hippocampal CA1 pyramidal neurons and have heterogeneous applied currents with a normal distribution. We derived a mean-field system for the network which consists of differential equations for the mean of the adaptation current and the synaptic conductance.

**Figure 1:**
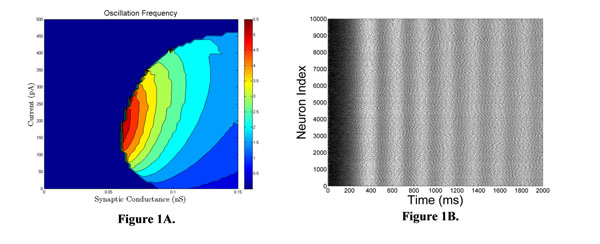
**A.** Mean-field prediction of the bursting frequency as a function of the unitary synaptic conductance and the mean applied current. **B.** Simulation of a network of 10,000 neurons with unitary conductance 0.058 nS and mean current 250 pA, showing an oscillation in the theta frequency range as predicted by the mean-field system of equations.

As CA1 is an area that displays prominent theta oscillations [4], we used the mean-field system to study how the frequency of bursting depends on various model parameters. Figure 1A shows an example study. These studies were successful in guiding numerical simulations of the large network. When parameter values determined from the mean-field analysis are used in a large network simulation, bursting of the predicted frequency occurs (Figure 1B).
